# Deep phenotyping as a contribution to personalized depression therapy: the GEParD and DaCFail protocols

**DOI:** 10.1007/s00702-023-02615-8

**Published:** 2023-03-23

**Authors:** Katharina Lichter, Catherina Klüpfel, Saskia Stonawski, Leif Hommers, Manuel Blickle, Carolin Burschka, Felix Das, Marlene Heißler, Anna Hellmuth, Jaqueline Helmel, Leonie Kranemann, Karin Lechner, Dominik Lehrieder, Amelie Sauter, Miriam A. Schiele, Vithusha Vijayakumar, Michael von Broen, Carolin Weiß, Caroline Morbach, Stefan Störk, Götz Gelbrich, Peter U. Heuschmann, Takahiro Higuchi, Andreas Buck, György A. Homola, Mirko Pham, Andreas Menke, Katharina Domschke, Sarah Kittel-Schneider, Jürgen Deckert

**Affiliations:** 1 Department of Psychiatry, Psychosomatics and Psychotherapy, Center of Mental Health, University Hospital of Würzburg, Margarete-Höppel-Platz 1, 97080 Würzburg, Germany; 2 Interdisciplinary Center for Clinical Research, University Hospital of Würzburg, Josef-Schneider-Str. 2, 97080 Würzburg, Germany; 3 Department of Clinical Research and Epidemiology, Comprehensive Heart Failure Center (CHFC), University Hospital of Würzburg, Am Schwarzenberg 15, 97078 Würzburg, Germany; 4 Department of Psychiatry and Psychotherapy, Faculty of Medicine, Medical Center-University of Freiburg, University of Freiburg, Hauptstr. 5, 79104 Freiburg, Germany; 5 Department of Medicine I, University Hospital of Würzburg, Oberdürrbacherstr. 6, 97080 Würzburg, Germany; 6 Institute of Clinical Epidemiology and Biometry, University of Würzburg, Josef-Schneider-Str. 2, 97080 Würzburg, Germany; 7 Clinical Trial Center, University Hospital of Würzburg, Würzburg, Germany; 8 Department of Nuclear Medicine, University Hospital of Würzburg, Oberdürrbacherstr. 6, 97080 Würzburg, Germany; 9 Dentistry and Pharmaceutical Sciences, Okayama University Graduate School of Medicine, Okayama, Japan; 10 Department of Neuroradiology, University Hospital of Würzburg, Josef-Schneider-Str. 2, 97080 Würzburg, Germany; 11 Department of Psychosomatic Medicine and Psychotherapy, Medical Park Chiemseeblick, Rathausstr. 25, 83233 Bernau am Chiemsee, Germany; 12 Department of Psychiatry and Psychotherapy, University Hospital, Ludwig Maximilian University of Munich, Nußbaumstr. 7, 80336 Munich, Germany; 13 Center for Basics in NeuroModulation, Faculty of Medicine, University of Freiburg, Breisacher Str. 64, 79106 Freiburg, Germany

**Keywords:** Major depressive disorder, Affective disorders, Predictive markers, Biomarkers, Brain–heart interaction

## Abstract

Depressive patients suffer from a complex of symptoms of varying intensity compromising their mood, emotions, self-concept, neurocognition, and somatic function. Due to a mosaic of aetiologies involved in developing depression, such as somatic, neurobiological, (epi-)genetic factors, or adverse life events, patients often experience recurrent depressive episodes. About 20–30% of these patients develop difficult-to-treat depression. Here, we describe the design of the GEParD (Genetics and Epigenetics of Pharmaco- and Psychotherapy in acute and recurrent Depression) cohort and the DaCFail (Depression-associated Cardiac Failure) case–control protocol. Both protocols intended to investigate the incremental utility of multimodal biomarkers including cardiovascular and (epi-)genetic markers, functional brain and heart imaging when evaluating the response to antidepressive therapy using comprehensive psychometry. From 2012 to 2020, 346 depressed patients (mean age 45 years) were recruited to the prospective, observational GEParD cohort protocol. Between 2016 and 2020, the DaCFail case–control protocol was initiated integrating four study subgroups to focus on heart-brain interactions and stress systems in patients > 50 years with depression and heart failure, respectively. For DaCFail, 120 depressed patients (mean age 60 years, group 1 + 2), of which 115 also completed GEParD, and 95 non-depressed controls (mean age 66 years) were recruited. The latter comprised 47 patients with heart failure (group 3) and 48 healthy subjects (group 4) of a population-based control group derived from the Characteristics and Course of Heart Failure Stages A–B and Determinants of Progression (STAAB) cohort study. Our hypothesis-driven, exploratory study design may serve as an exemplary roadmap for a standardized, reproducible investigation of personalized antidepressant therapy in an inpatient setting with focus on heart comorbidities in future multicentre studies.

## Introduction

Depressive episodes in uni- and bipolar-affective disorders are multifactorial comprising (neuro-)biological and psychosocial factors. Such episodes can affect people during their entire life span compromising life quality and expectancy from early on (Otte et al. 2016; Vieta et al. 2018; Solmi et al. 2022). While the clinical phenomenology in depressed patients varies within a known framework of symptoms and may turn into a chronic, treatment-resistant condition in about 20–30% of the individuals (Fava and Davidson 1996), the neurobiological analogues of depression are heterogeneous and less well defined. Regarding personalized antidepressant therapy, the translation into clinical biomarkers for (deep) phenotyping of depressed patients has remained challenging.

The investigation of genetic heritability and gene–environment interactions (Karg and Sen 2012) based on candidate genes of neurotransmitter systems (Caspi et al. 2003; Baune et al. 2008) has contributed to the development of concepts on interacting risk and disease-modifying factors of depression. The read-out of epigenetic modifications ‘picturing’ a patient’s (adverse) life events has allowed to study pharmacoepigenetics patterns for the prediction of an impaired response to treatment, e.g. with selective serotonin reuptake inhibitors (SSRIs) (Schiele et al. 2021). (Epi-)genome-wide association studies (E-/GWAS) in depression, e.g. (Major Depressive Disorder Working Group of the Psychiatric Genomic Consortium et al. 2013; Menke et al. 2012a, b; Okbay et al. 2016; Story Jovanova et al. 2018) have upscaled single nucleotide polymorphisms (SNP) investigations to larger cohorts, thereby defining further genetic regions of interest and facilitating novel (predictive) measurements such as polygenic risk scores (Fanelli et al. 2022). Recent GWAS for depression have essentially emphasized the importance of genes in synaptic structure and function (Howard et al. 2019). Most of these studies, however, focused on categorical definitions of diseases not considering that depression is a heterogenous, dimensional, and systemic condition affecting multiple organs of the body (Sotelo and Nemeroff 2017).

To define prospective, reproducible biomarkers for depression subtypes based upon whole body disease concepts, a standardized organ and/or body system-tailored deep phenotyping for large cohorts of depressed patients is instrumental.

Essential stress-based pathophysiological mechanisms of depression comprise an impairment of the hypothalamus–pituitary–adrenal (HPA) axis, the adrenergic autonomic nervous system (ANS), and the immune system (Carney et al. 2005; Pariante and Lightman 2008; McEwen and Akil 2020; Beurel et al. 2020). In the pathogenesis of depression, a modulated heart–brain interaction may be responsible for the elevated risk of cardiovascular disease and cardiac mortality in individuals with major depression (Nemeroff and Goldschmidt-Clermont 2012; Hare et al. 2014; Nielsen et al. 2021). Previous studies showed that dysregulation of the HPA axis in depression is linked to an impaired sensitivity of glucocorticoid receptors (GR) (Pariante and Lightman 2008; Menke et al. 2012a, b). Depressed patients are at higher risk for cardiovascular diseases (CVD) including heart failure (Gustad et al. 2014), possibly due to a dysfunctional adrenergic ANS. This may result in increased heart rate, hypertension, and a reduced heart rate variability (HRV) (Koch et al. 2019), i.e. symptoms shared with patients suffering from heart failure (Parati and Esler 2012). Even though the insular cortex—a brain region associated with interoceptive attention (Wang et al. 2019) which is compromised in depressed patients (Eggart et al. 2019)—and its functional networks are considered the neuronal representations of ANS activity (Beissner et al. 2013), it remains unclear whether and how these neuronal substrates may affect the course of patients with depression and heart failure, respectively. Genotyping in patients with heart failure and comorbid depression revealed that genetic variants implicated in anxious behaviour (*NPSR1*) (Angermann et al. 2017) as well as inflammation (*C-reactive protein* (*CRP)*, *interleukin 6* (*IL-6)*) (Kittel-Schneider et al. 2018) modify the risk of progression and mortality. Patients with heart failure, on the other hand, not only face a higher risk for depression (Rutledge et al. 2006), but also an increased mortality caused by depression itself (Penninx et al. 2001; Zambrano et al. 2020). However, patients with heart failure and comorbid depression experience no prognostic benefit from antidepressive pharmacological treatment, indicating heterogeneity of the depression phenotype (Angermann et al. 2016).

Here, we introduce the design of the observational GEParD (Genetics and Epigenetics of Pharmaco- and Psychotherapy in acute and recurrent Depression) cohort and the DaCFail (Depression associated Cardiac Failure) protocol, the latter designed as a tailored protocol on the role of stress systems in heart–brain interaction. The main intention was to monitor a naturalistic antidepressant therapy response within an inpatient setting applying a broad repertoire of psychometric and neuropsychological testing, routine blood diagnostics including endocrine and inflammatory biomarkers, established genetic and epigenetic testing, and finally a comprehensive cardiac phenotyping including parameters of the ANS. We finally implemented our ideas in an exploratory approach to generate hypotheses for follow-up studies dealing among others with the following questions:

1. Which psychometric parameters and stress-related biomarkers (endocrine, inflammatory, and ANS) can be defined as biomarkers of depression symptoms and antidepressant treatment response?
2. Which (epi-)genetic biomarkers of depression symptoms and antidepressant treatment response can be defined in interaction with proximal and/or distal life events?
3. Which parameters of the heart–brain interaction differentiate depression with and without heart failure as compared to healthy controls and/or are relevant for antidepressant treatment response?
4. How does the function of the insula and its networks differ in depressed patients with and without heart failure as compared to healthy controls?

## Methods

### The protocols

**Fig. 1:**
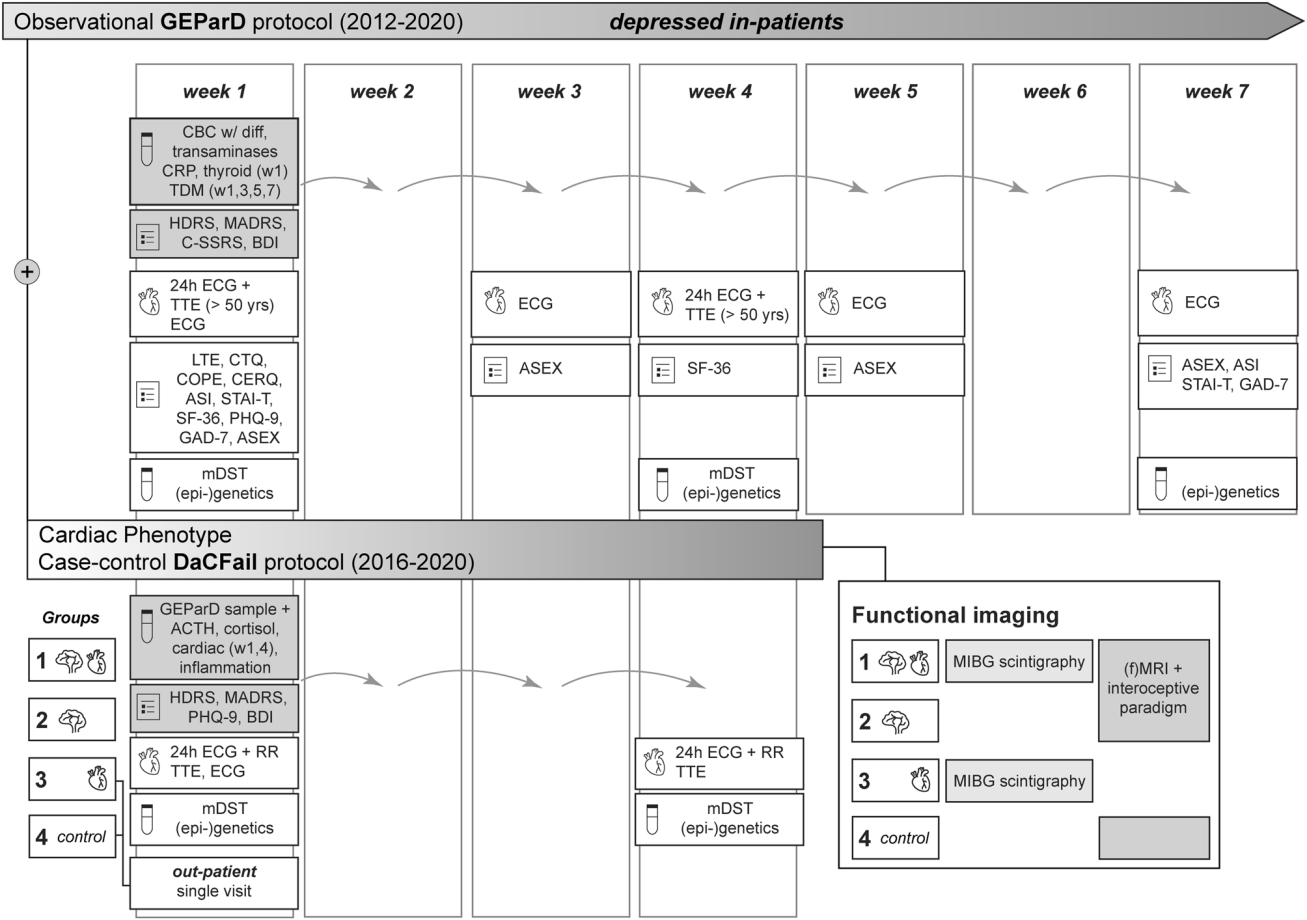
Study designs. After study enrolment, patients were scheduled for weekly blood sample collection in both study protocols. Psychometry was carried out within the clinical routine, with exemptions. In selected study weeks, patients were scheduled for cardiac assessment, modified dexamethasone-suppression test (mDST), and blood sample collection for (epi-)genetic analyses. Patients of DaCFail group 1 and 3 underwent MIBG scintigraphy. Abbreviations: *diff* = differential, *MIBG* = ^123^I-meta-iodobenzylguanidine, *RR* = blood pressure, *TDM* = therapeutic drug monitoring, *TTE* = transthoracic echocardiography, *w* = week, *w/* = with. For psychometry and multimodal biomarkers including functional imaging in detail, please see Tables 2 and 3

The GEParD protocol was initiated in 2012 and carried out as a prospective observational cohort study of hospitalized, depressed uni- and bipolar patients with weekly measurements for up to 7 weeks (Fig. 1). In a second phase (2016–2020), the DaCFail protocol was initiated complementing the existing study concept by parameters focusing on the stress and the cardiac system (Fig. 1). The DaCFail protocol was designed as a refined case–control protocol for 200 patients and methodologically based on GEParD. For DaCFail, patients were enclosed in four study groups: Group 1, patients with depression and heart failure; Group 2, depressed patients; Group 3, patients with heart failure; Group 4, healthy subjects (control group, Table 1). Measurements were carried out weekly for a maximum of four weeks duration (Fig. 1). Both functional magnetic resonance imaging (fMRI) of the insular cortex and ^123^I-meta-iodobenzylguanidine (MIBG) scintigraphy were integrated as quantitative imaging modalities within the DaCFail study protocol (Fig. 1). The procedures were approved by the local ethics committee of the University Hospital of Würzburg (GEParD: vote no 104/12 and 128/15, DaCFail: vote no. 285/14) and were carried out in accordance with the ethical standards of the Declaration of Helsinki and its later amendments (Williams 2013).

**Table 1:** Inclusion and exclusion criteria for the GEParD and DaCFail protocols

(i) Inclusion criteria	
*GEParD* Age of 18–80 yrs Clinically phenotypical depressive disorder (DSM-IV)	*DaCFail* Age ≥ 50 yrs *Group 1*: depressive episode (DSM-IV, HDRS ≥ 14) + LVEF < 52% ♂ / 54% ♀ *Group 2*: depressive episode (DSM-IV, HDRS ≥ 14) + normal LVEF *Group 3*: no depression, LVEF < 52% ♂ / 54% ♀ *Group 4*: no depression + normal LVEF (healthy control probands from the STAAB cohort)
(ii) Exclusion criteria	
*GEParD* Inability to give written informed consent, presence of a depressive disorder caused by substance use disorder, severe neurological condition, e.g. Parkinson’s disease, dementia, or stroke, malignant tumours, diagnosis of schizophrenia/psychosis, systemic medication with glucocorticoids	*DaCFail* Inability to give written informed consent, presence of a depressive disorder caused by substance use disorder, severe neurological condition, e.g. Parkinson’s disease, dementia, or stroke, malignant tumours, diagnosis of schizophrenia/psychosis, systemic medication with glucocorticoids *Group 3 and 4*: Current or past depressive episode (PHQ-9, SCID-I), substance use disorder

*DSM-IV* Diagnostic and Statistical Manual Of Mental Disorders-IV, *HDRS* Hamilton Depression Scale, *LVEF* left ventricular ejection fraction, *PHQ-9* Patient Health Questionnaire, *SCID-I* Structured Clinical Interview for DSM-IV axis I disorders, *STAAB* characteristics and course of heart failure stages A–B and determinants of progression (Wagner et al. 2017)

### Participant selection criteria

The GEParD protocol recruited patients aged 18–80 years (Table 1) presenting with a unipolar and bipolar depressive disorder diagnosed by a validated, standardized interview according to DSM-IV criteria (Structured Clinical Interview for DSM, SCID-I (First and Gibbon 2004)). Antidepressant treatment was carried out according to the treating physician’s choice within an inpatient setting using psychopharmacology, psychotherapy, and electroconvulsive therapy (ECT).

Within the DaCFail protocol, subjects beyond an age of 50 years were eligible for one of the following study arms applying the selection criteria detailed in Table 1. Healthy controls without medical and mental illness precondition were asked for participation from the population-based “Characteristics and Course of Heart Failure Stages A–B and Determinants of Progression (STAAB)” cohort study, comprising a representative age-stratified sample of about 5000 Würzburg residents aged 30–79 years at baseline assessment (Morbach et al. 2021; Wagner et al. 2017). Healthy subjects were matched in age (≥ 50 years) and gender to the other study groups. For both the GEParD and/or DaCFail study protocol, the exclusion criteria listed in Table 1 were applied.

### Recruitment strategy

**Fig. 2:**
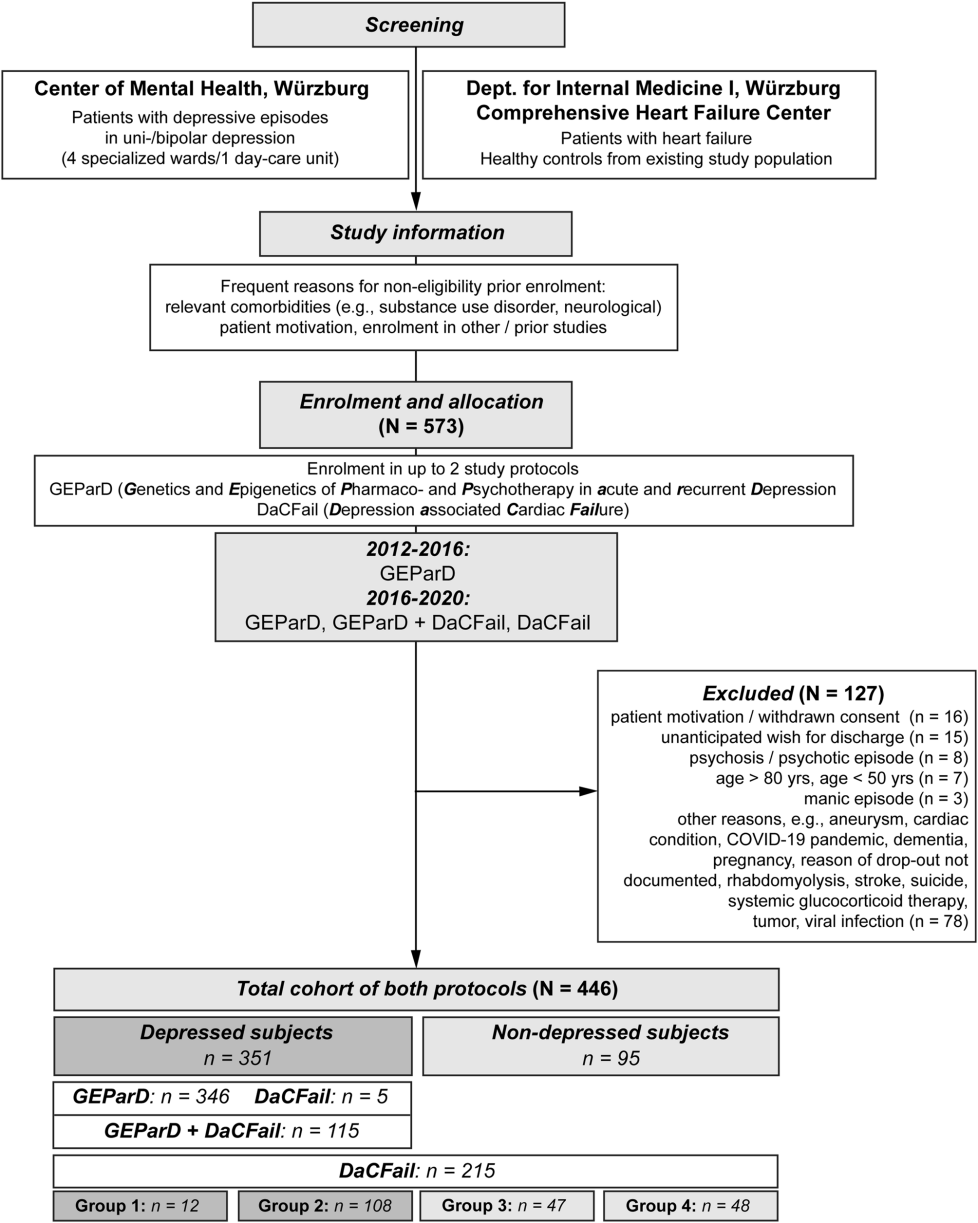
Consort chart for the GEParD and DaCFail protocols (2012–2020). Overview of screening and enrolment process in the GEParD and DaCFail study protocols

For both study protocols, hospitalized depressed patients were recruited on-site by specialized staff within the inpatient setting of the Department of Psychiatry, Psychotherapy and Psychosomatic Medicine, University Hospital Würzburg, Germany. Here, four wards with a therapy focus on affective disorders and intensive psychotherapy as well as one neuropsychiatric day-care unit specialized for geriatric patients offer a naturalistic study environment (Fig. 2). Newly admitted patients fulfilling the inclusion criteria of the study protocols were informed about a possible participation by their attending physician. Detailed information sheets of the study protocols were provided with the aim of informing about the study course, study criteria, and applied procedures and measurements. For the patients diagnosed with heart failure (DaCFail Group 3) and the healthy controls (DaCFail Group 4), the recruitment was performed in cooperation with the Department of Internal Medicine I and the Comprehensive Heart Failure Center Würzburg (CHFC) (Fig. 2)*.* For both groups, study assessments were carried out at the Center of Mental Health.

### Minimal recruitment number

The GEParD study protocol as a prospective observational study aimed to recruit 500 depressed patients. For the DaCFail study protocol, the minimum recruitment number of participants was defined by a power analysis for expected HRV differences between groups as the original primary outcome of the case–control design. To test on HRV differences in depressed patients and controls with an analysis power of 0.8 on a significance level of *α* = 0.05 and an effect size of Cohen’s *d* = 1, a minimum recruitment number of *N* = 24 participants per group was calculated. It was assumed that investigated effects differ in the range of one standard deviation (SD) in the cohort of depressed patients versus controls. Given the four study groups, variances were doubled leading to a minimal recruitment number of *N* = 48 participants for each of the four DaCFail study groups under the defined test conditions.

### Psychometric and neuropsychological testing

In the process of and after study enrolment, psychometric evaluation of patients was carried out in scheduled study rounds within the inpatient setting or single appointments at the outpatient clinic (DaCFail Group 3, 4) (Fig. 1, Table 2).

**Table 2:** Psychometry, life events, and coping style

(i) Screening tools for depression, anxiety, suicidal ideation, and life quality
Depression module of the Patient Health Questionnaire (PHQ-9) (Kroenke et al. 2001) 7-Item anxiety scale for generalized anxiety disorder (GAD-7) (Spitzer et al. 2006) Short-Form-36 Health Survey (SF-36) (Bullinger et al. 1995) The Structured Clinical Interview for DSM-IV Axis I Disorders (SCID-I) (First and Gibbon 2004)
(ii) Dimension of depressive episodes
Anxiety Sensitivity Index (ASI) (Reiss et al. 1986) Columbia-Suicide Severity Rating Scale (C-SSRS) (Posner et al. 2011) Beck Depression Inventory (BDI) (Beck et al. 1961) Hamilton Depression Scale (HDRS) (Hamilton 1960) Montgomery Ǻsberg Depression Rating Scale (MADRS) (Montgomery and Asberg 1979) State-Trait Anxiety Inventory (STAI-T)
(iii) Neurocognition
Cognitive Emotion Regulation Questionnaire (CERQ) (Garnefski et al. 2001) Montreal Cognitive Assessment (MOCA) (Nasreddine et al. 2005)
(iv) Life events and coping strategies
*Distal and proximal life events* Childhood Trauma Questionnaire (CTQ) (Bernstein et al. 2003) List of Threatening Experiences (LTE) (Brugha et al. 1985) *Stress system* Coping Orientation to Problems Experienced Inventory (COPE) (Carver et al. 1989) *Life quality* Arizona Sexual Experience Scale (ASEX) (McGahuey et al. 2000)

As screening tool for depressive and anxious symptoms, the SCID-I, the depression module of Patient Health Questionnaire-9 (PHQ-9) and the 7-item anxiety scale for generalized anxiety disorder (GAD-7) (Spitzer et al. 2006) were used (Table 2).

Depression severity and dimension were assessed by the Beck Depression Inventory (BDI-II) (Beck et al. 1961), Hamilton Depression Rating Scale (HDRS_21_, 21 items in total, 17 of 21 items were used for evaluation) (Hamilton 1960), and the Montgomery Ǻsberg Depression Rating Scale (MADRS) (Montgomery and Asberg 1979), further, particularly focusing on the entity of anxious depression, the Anxiety Sensitivity Index (ASI) (Reiss et al. 1986) and the State-Trait Anxiety Inventory (STAI-T) were used. Emotional regulation in patients was evaluated by the Cognitive Emotion Regulation Questionnaire (CERQ) (Garnefski et al. 2001). The Montreal Cognitive Assessment (MOCA) (Nasreddine et al. 2005) was used to assess possible neurocognitive impairments.

To contextualize gene–environment interactions, distal and proximal life events of patients were evaluated by the Childhood Trauma Questionnaire (CTQ) (Bernstein et al. 2003) and the List of Threatening Experiences (LTE) (Brugha et al. 1985). The patient’s coping strategies were assessed by the Coping Orientation to Problems Experienced Inventory (COPE) (Carver et al. 1989). The Columbia-Suicide Severity Rating Scale (C-SSRS) (Posner et al. 2011) was used to evaluate suicidal ideation (Menke et al. 2012a, b). The Short-Form-36 Health Survey (SF-36) (Bullinger et al. 1995) assessed the life quality of patients with the Arizona Sexual Experience Scale (ASEX) (McGahuey et al. 2000) monitoring sexual dysfunction.

### Physical activity

To monitor (altered) locomotor activity in depressed patients (Wuthrich et al. 2022) and to investigate possible interactions with the HPA axis (Menke et al. 2014), step counts were quantified in a sub-cohort of depressed patients using actigraphy (ActiGraph GT9X Link, ActiGraph LLC, Pensacola, US).

### Blood sampling

All enrolled patients were scheduled for blood sampling at defined time points to perform routine blood analysis including established therapeutic drug monitoring (TDM) of psychopharmaceutical medication, analysis of endocrine and inflammation markers, as well as genetic and epigenetic analysis (Fig. 1, Table 3). Blood sample collection was carried out by trained staff.

**Table 3:** Laboratory, cardiological phenotype, and imaging

Biomaterial asservation	Method	Parameter
(i) *Laboratory*		
SST, EDTA	Standard haematology assay	CBC w/ differential, GOT, GPT, γ-GT, CRP
SST	Standard haematology assay	NTproBNP, trop T/I (cardiac)
SST	Enzyme-linked immunosorbent assay	IL-6, IL-1, TNF-α (inflammation)
SST		TSH, fT3, fT4 (thyroid)
SST	mDST	ACTH, cortisol
SST	TDM using high-performance liquid chromatography	Psychopharmaceutical drug concentration
EDTA	Sequencing	DNA methylation, genotyping
PAXgene^™^ blood tube	RNA extraction, quantitative real-time polymerase chain reaction	mRNA expression
(ii) *Quantitative cardiological assessment*		
	24-h ECG	bpm, HRV (SDNN, SDANN, SDRR, pNN50, RMSSD), VES, SVES
	24-h blood pressure	RR_sys_, RR_dia_
	Echocardiography	LVEF, IVS, LVPWd/s, LVEDD
	Actigraphy	Step count
	MIBG scintigraphy	HMR
(iii) *Neuronal morphometry and interoception*		
	MRI	High-resolution T1-weighted imaging (of the whole brain including the insular cortex)
	mod. Schandry task	Score considering counted and recorded heartbeats
	rs-fMRI	Functional connectivity analysis via resting-state fMRI

*IVST* interventricular septum thickness, *LVPWd/s* left ventricular posterior wall end diastole and end systole, *LVEDD* left ventricular end-diastolic diameter, *pNN50* percentage of successive RR intervals that differ by more than 50 ms, *RMSSD* root mean square of successive RR interval differences, *rs* resting state, *SDNN* standard deviation (SD) of NN intervals, *SDANN* SD of the 5 min average NN intervals, *SDRR* SD of RR intervals, *SST* serum separator tube, *SVES* supraventricular extrasystole, *VES* ventricular extrasystole, *w/* = with. Other abbreviations are stated in the text

On a weekly basis until study week 4 (DaCFail) and study week 7 (GEParD), respectively, a routine blood sample of each patient was collected for analysis of the complete blood count (CBC) with differential blood cell count, transaminases (glutamic oxaloacetic transaminase (GOT), glutamate-pyruvate transaminase (GPT), γ-glutamyltransferase (γ-GT)), and CRP. In study week 1, thyroid markers (thyroid-stimulating hormone (TSH), free triiodothyronine (fT3), and free thyroxine (fT4)) were collected. In study week 1 and 4 (DaCFail), blood samples to investigate the inflammatory parameters IL-6, interleukin 1 (IL-1), and tumour necrosis factor alpha (TNF-α) were additionally collected. For analyses of (epi-)genetic parameters and the HPA axis, please see the respective sections.

### (Epi-)genetic analyses

In study week 1, 4 and 7 as well as parallel to the modified dexamethasone-suppression test (mDST), PAXgene^™^ blood test tubes (Qiagen, Hilden, Germany) for analysis of mRNA expression and ethylene diamine tetra-acetic acid (EDTA) tubes for analyses of DNA methylation and analysis of genetic variants were taken.

### Modified dexamethasone-suppression test (mDST)

An mDST, as previously described (Menke et al. 2021; Leistner and Menke 2018), was applied at week 1 and 4 in inpatients and once in the above described outpatient setting (Fig. 1, Table 3). Before oral administration of 1.5 mg dexamethasone, blood was drawn by trained staff at 6 p.m. for the analysis of a CBC with differential blood cell count, cortisol, and adrenocorticotropic hormone (ACTH). PAXgene^™^ blood test tubes (Qiagen, Hilden, Germany) for analysis of mRNA expression, and EDTA tubes for analysis of DNA methylation and genetic variants were also used. Three hours post-medication, a second blood sample was collected for analysis of the above-mentioned parameters.

### Assessment of cardiovascular parameters

Comprehensive cardiological diagnostics were performed for all patients enclosed in the DaCFail study protocol. This included 24 h electrocardiogram (ECG), 24 h blood pressure measurement (Riva-Rocci, RR), and transthoracic echocardiography (TTE) for patients ≥ 50 yrs (Table 3). In study week 1 and 4 of the DaCFail study protocol (Fig. 1), amino-terminal pro-hormone brain natriuretic peptide (NTproBNP), and troponin T/I (trop T/I) were collected as cardiac blood biomarkers.

### ^123^I-meta-iodobenzylguanidine scintigraphy

MIBG scintigraphy (Chirumamilla and Travin 2011) was used to monitor synaptic activity of cardiac sympathetic neurons in HF patients (DaCFail Group 1, 3) and carried out at the Department for Nuclear Medicine at University Hospital of Würzburg (Werner et al. 2018). Increased uptake of the tracer ^123^I-MIBG in synaptic vesicles was used as a marker of synaptic transmission and plasticity in sympathetic neurons and quantified via the heart mediastinum ratio (HMR). The imaging protocol set two time points of scanning (early scan 15 min post-injection, delayed scan 4 h post-injection). For single- and three-dimensional imaging, a planar scintigraphy protocol as well as single-photon emission computed tomography (SPECT) was used.

### Functional MRI and interoceptive paradigm

For an investigation of the insular cortex–heart axis in depression (Fig. 1, Table 3), functional MRI measurements in combination with interoceptive paradigms were applied. The insular cortices are considered as neuronal substrates for the perception of internal sensory information (interoception), which is discussed to be compromised in patients with depression (Eggart et al. 2019), as well as of ANS activity (Beissner et al. 2013). Patients of DaCFail groups suffering from depression (groups 1 + 2) and controls (group 4), respectively, underwent the following measurements in one single session:

(i) Based on the concept of interoceptive accuracy (Garfinkel et al. 2015), a modified version of the Schandry task (Schandry 1981) as heartbeat perception task was used to objectively measure perception of internal sensory information.
(ii) HRV recordings were established to evaluate possible confounder phenomena and analysed for a defined time (300 s) using the RMSSD (Root Mean Sum of Squared Distance, Unit: ms).
(iii) For morphometry of defined regions of interest in the insular cortex of both hemispheres (ventral and dorsal anterior insula, posterior insula) and for functional connectivity analyses, MRI data were acquired on a 3 Tesla scanner (MAGNETOM Skyra, Siemens, Erlangen, Germany). A 64-channel head coil was used. Structural images were acquired with a high-resolution T1-weighted magnetization prepared rapid gradient echo (MPRAGE). Resting-state recordings were acquired for a total duration of 10 min using T2*-weighted blood oxygen level-dependent (BOLD) images as echo-planar imaging (EPI) sequence. Participants were instructed to stay awake and keep their eyes open.

### Study end point

For both study protocols, at the study end point after 7 weeks or after 4 weeks of antidepressant therapy in the inpatient setting, the difference of the individual HDRS scores was measured (Fig. 1). This therapy outcome parameter in both study protocols was identical because the second case-controlled study protocol (DaCFail) had been designed on the initial prospective, observational GEParD cohort protocol. Demographic characterization, psychometric parameters, somatic, laboratory, and (epi-)genetic biomarkers were used for evaluation of and/or correlation with therapy response. In the DaCFail study protocol, patients in addition were evaluated by HRV analyses (24 h ECG) using the RMSSD as the original primary outcome parameter and HMR analyses using MIBG scintigraphy in correlation with psychometric measurements of depression.

### Statistics

All statistical calculations were and will be carried out with the IBM SPSS software package 26 (SPSS Inc., Chicago, USA) and SigmaPlot 14 (Systat Software, Düsseldorf, Germany). Further calculations may implement custom-written programming scripts for individual deep phenotyping based on (un-)supervised machine leaning algorithms.

## Results

### Recruitment

In total, *N* = 573 patients were recruited to the GEParD study protocol between October 2012 and December 2020 (*N* = 458 patients) and to the DaCFail study protocol between March 2016 and December 2020 (*N* = 268 patients). For the parallel inclusion in both protocols, *N* = 153 patients were initially evaluated. In the process of enrolment in one or both protocols, *N* = 127 patients had to be excluded due to somatic conditions or unforeseen disease courses (*N* = 94), changes in the patients’ personal motivation for study participation (*N* = 16), and unanticipated wishes for discharge (*N* = 15). In most cases, somatic or mental exclusion criteria (manic or psychotic episodes) had been unknown and/or unfolded within the first study week based upon the detailed diagnostic pipeline. Of the 120 participating depressed patients in the DacFail protocol, *N* = 115 completed the GEParD study protocol in parallel. In the process of and after enrolment, five depressed patients decided to solely participate in the DaCFail protocol. The number of patients completing one or both protocols thus consisted of 446 probands, of which 351 patients suffered from depression and 95 probands, recruited for the DaCFail protocol, were considered as non-depressed controls (patients with heart failure, healthy probands). Sizes of study groups are further detailed in Fig. 2 and Tables 4 and 5.

**Table 4:** Basic demographic data and baseline depressive phenotype for the entire cohort of depressed patients (*N* = 351)

Parameter	Reference	Depressed patients			
		Total (*N* = 351)		GEParD (*N* = 346)	
*Baseline demographic data*					
		Mean ± SD (range)		Mean ± SD (range)	
Age [yrs]	18–80	45.8 ± 15.3 (18–80)		45.6 ± 15.3 (18–80)	
		*N*	%	*N*	%
Gender	m/f	149/202	42.5/57.5	146/200	42.2/57.8
Marital status	Married	163	46.4	162	46.8
	Single	93	26.5	93	26.9
	Relationship	37	10.5	37	10.7
	Separated/divorced	40	11.4	39	11.3
	Widowed	16	4.6	15	4.3
	Missing	2	0.6	-	-
Education	Apprenticeship/training	225	64.1	222	64.2
	College/university	57	16.2	57	16.5
	None	64	18.2	64	18.5
	Missing	5	1.4	3	0.9
Smoking	y/n/missing	123/119/109	35.0/33.9/31.1	123/115/108	35.5/33.2/31.2
Alcohol	y/n/missing	49/133/169	14.0/37.9/48.1	46/131/169	13.3/37.9/48.8
*Baseline depressive phenotype*					
Depressive episode w/o psychotic symptoms	First depressive episode	49	14.0	48	13.9
	Recurrent depressive disorder	259	73.8	255	73.7
	Bipolar disorder	43	12.3	43	12.4
N of depressive episodes (incl. current)	1	52	14.8	52	15.0
	2–3	92	26.2	92	26.6
	4–5	49	14.0	49	14.2
	> 5	71	20.2	71	20.5
	Not clearly definable	70	20.0	70	20.2
	Not stated	17	4.8	12	3.5
Age of first onset [yrs]	< 18	75	21.4	75	21.7
	18–29	95	27.1	95	27.5
	30–39	54	15.4	54	15.6
	40–49	73	20.8	73	21.1
	50–59	34	9.7	34	9.8
	> 60	8	2.3	8	2.3
	Not stated	12	3.4	7	2.0
Family history for depression	y/n/missing	222/123/6	63.2/35.0/1.7	222/123/1	64.2/35.5/0.3
Previous treatment with AD	y/n/missing	286/53/12	81.5/15.1/3.4	286/53/7	82.7/15.3/2.0
History of suicide attempt(s)	y/n/missing	84/259/8	23.9/73.8/2.3	84/259/3	24.3/74.9/0.9
Sleep disturbances	y/n/missing	251/100/0	71.5/28.5/0	247/99/0	71.4/28.6/0
*Baseline questionnaires for depression (admission, w1)*					
		Mean ± SD/median (25th–75th percentile)		Mean ± SD/median (25th–75th percentile)	
HDRS		21.9 ± 6.6		20.3 ± 6.0	
BDI-II		24 (17–32.5), *N* = 344		24.0 (17–33), *N* = 339	
MADRS		32 (27–36), *N* = 252		32 (27–36), *N* = 247	
GAD-7		12 (8–15), *N* = 248		12 (8–15), *N* = 243	

*AD* antidepressant, *BDI-II* Beck Depression Inventory II, *GAD-7* 7-item anxiety scale for generalized anxiety disorder, *HDRS* Hamilton Depression Scale, *N/n* sample number, *MADRS* montgomery Ǻsberg depression rating scale, *PHQ-9* Patient Health Questionnaire-9, *SD* standard deviation, *w1* study week 1, *yrs* years

**Table 5:** Basic demographic data and baseline depressive phenotype for the DaCFail study groups (*N* = 215)

Parameter	Reference	Depressed patients				Non-depressed patients			
		DaCFail Gr 1 *N* = 12		DaCFail Gr 2 *N* = 108		DaCFail Gr 3 *N* = 47		DaCFail Gr 4 *N* = 48	
		Mean ± SD (range)		Mean ± SD (range)		Mean ± SD (range)		Mean ± SD (range)	
*Baseline demographic data*									
Age [yrs]	≥ 50	62.1 ± 10.1 (51–79)		59.5 ± 7.7 (50–80)		69.2 ± 8.3 (52–84)		63.1 ± 6.3 (51–81)	
		N	%	N	%	N	%	N	%
Gender	m/f	6/6	50/50	40/68	37/63	41/6	87.2/12.8	25/23	52.1/47.9
Marital status	Married	5	41.7	63	58.3	34	72.3	34	70.8
	Single	0	0	8	7.4	3	6.4	0	0
	Relationship	1	8.3	9	8.3	0	0	4	8.3
	Separated/divorced	1	8.3	19	17.6	0	0	6	12.5
	Widowed	3	25.0	9	8.3	0	0	4	8.3
	Missing	2	16.7	0	0	10	21.3	0	0
Education	Apprenticeship/ training	10	83.3	71	65.7	32	68.1	22	45.8
	College/ university	0	0	17	15.7	5	10.6	23	47.9
	None	0	0	18	16.7	0	0	1	2.1
	Missing	2	16.7	2	1.9	10	21.3	2	4.2
Smoking	y/n/missing	2/8/2	16.7/66.7/16.7	29/66/13	26.9/61.1/12.0	2/44/1	4.3/93.6/2.1	6/42/0	12.5/87.5/0
Alcohol	y/n/missing	5/6/1	41.7/50/8.3	24/65/19	22.2/60.2/17.6	20/24/3	42.6/51.1/6.4	26/4/18	54.2/8.3/37.5
*Baseline depressive phenotype*									
Depressive episode w/o psychotic symptoms	First depressive episode	1	8.3	12	11.1	n/a			
	Recurrent depressive disorder	10	83.3	81	75.0				
	Bipolar disorder	1	8.3	15	13.9				
N of depressive episodes (incl. current)	1	0	0	9	8.3	n/a			
	2–3	2	16.7	28	25.9				
	4–5	2	16.7	16	14.8				
	> 5	3	25.0	40	37.0				
	Not clearly definable	0	0	15	13.9				
	Not stated	5	41.7	0	0				
Age of first onset [yrs]	< 18	1	8.3	13	12.0	n/a			
	18–29	1	8.3	18	16.7				
	30–39	2	16.7	17	15.7				
	40–49	1	8.3	32	29.6				
	50–59	1	8.3	21	19.4				
	> 60	1	8.3	2	1.9				
	Not stated	5	41.7	5	4.6				
Family history for depression	y/n/missing	2/5/5	16.7/41.7/41.7	69/39/0	63.9/36.1/0	n/a			
Previous treatment with ADs	y/n/missing	4/3/5	33.3/25.0/41.7	96/12/0	88.9/11.1/0	n/a			
History for suicide attempt(s)	y/n/missing	0/7/5	0/58.3/41.7	24/82/2	22.2/75.9/1.9	n/a			
Sleep disturbances	y/n/missing	9/3/0	75/25/0	79/29/0	73.1/26.9/0	12/34/1	25.5/72.3/2.1	11/37/0	22.9/77.1/0
*Baseline questionnaires for depression / screening tools (admission, w1)*									
		Median (25th–75th percentile)		Median (25th–75th percentile)		Median (25th–75th percentile)		Median (25th–75th percentile)	
HDRS		18.5 (9.5–29.25)		22.5 (18–27)		2 (1–4)		1 (0–2.75)	
BDI-II		17 (11–28.25)		21 (14–30), *N* = 107		6 (4–9)		6 (5–8)	
MADRS		25.5 (12.5–35.75)		30 (26–35)		2 (0–3)		0 (0–1)	
GAD-7		6.5 (4.25–16)		11 (7–15), *N* = 107		1 (0–4)		1 (0–1.75), *N* = 39	
PHQ-9		11 (7–20.5), *N* = 9		14 (10–19), *N* = 107		2 (0–3.25), *N* = 46		2 (0.25–3), *N* = 39	

*AD* antidepressant, *BDI-II* Beck Depression Inventory II, *GAD-7* 7-item anxiety scale for generalized anxiety disorder, *HDRS* Hamilton Depression Scale, *MADRS* Montgomery Ǻsberg Depression Rating Scale, *N/n* sample number, *PHQ-9* Patient Health Questionnaire-9, *SD* standard deviation, *w1* study week 1, *yrs* years

### Demographics

The mean age of all 351 depressed subjects was 45.8 years (standard deviation (SD) 15.3, range 18–80), *N* = 202 depressed participants (57.5%) were male. *N* = 200 depressed patients (57.0%) were married or engaged in a relationship. The control cohort of 95 non-depressed subjects had a mean age of 66.1 years (SD 8.0, range 51–84), of which 66 participants (69.5%) were male. *N* = 72 control patients (75.8%) were married or engaged in a relationship. Approximately, one-third of the depressive participants (*N* = 123, 35.0%) were active smokers. Basic demographic data of the different study groups including the DaCFail subgroups are further detailed in Tables 4 and 5.

### Baseline depressive phenotype

The cohort of depressed patients (*N* = 351) consisted of 305 unipolar depressed patients, of which 49 patients (14.0%) suffered from their first depressive episode and 259 patients (73.8%) from a recurrent depressive episode (basic characteristics of all depressed patients are stated in Table 4). *N* = 43 patients (12.3%) had the diagnosis of a depressive episode within a bipolar-affective disorder. Regarding the disease load of depressive episodes, *N* = 120 patients (34.2%) stated an experience of more than three depressive episodes before study enrolment. In the total cohort, two peaks of first depression onset could be differentiated in patients retrospectively based on the previous medical history at the time point of study enrolment: 1. youth and adolescence (< 18 years) and young adulthood (19–29 years) in 170 patients (48.4%); 2. fifth decade of life (40–49 years) in 73 patients (20.8%). Before admission, 286 patients (81.5%) had received treatment with antidepressants. Nearly 25% of all depressed patients had a history of suicide attempts. Most depressed patients (71.5%) had suffered from various sleep disturbances. Regarding the initial quantitative psychometry in the first week of study, depressed patients were assessed by researchers with a mean of 22 (SD 6.6) points in the HDRS (17 of 21 items used for evaluation) and a median of 32 (27–36) points in the MADRS. Using the BDI-II, patients self-rated the severity of their depressive episode with a median of 24 points (16–32). 70.7% of the depressed patients suffered from anxious symptoms and rated a median of 12 points (SD 8–15) on the GAD-7. Further data on the basic depressive phenotype of the DaCFail groups are detailed in Table 5.

Regarding their baseline somatic phenotype, the majority (*N* = 212, 60.4%) of depressive participants were classified as overweight or obese (body mass index (BMI) ≥ 25). In the 24 h RR analyses, nearly a third of all depressed patients suffered from either a stage of prehypertension with elevated measurements in total (RR_sys_ 130–139 mmHg and/or RR_dia_ 85–89) or from arterial hypertension at a minimum stage of 1 (stage 1: RR_sys_ 140–159 mmHg and/or RR_dia_ 90–99 mmHg, stage 2: RR_sys_ 160–179 mmHg and/or RR_dia_ 100–109 mmHg).

## Discussion

The GEParD cohort study as prospective observational study protocol and the DaCFail study as a case–control study protocol are an example for deep phenotyping of depressed patients in an inpatient setting, particularly emphasizing the stress system and the heart–brain axis. The present repertoire of psychometry, tailored quantification of somatic and cardiac parameters, as well as laboratory parameters including (epi-)genetic analyses allows to evaluate response or resistance to antidepressant therapy during hospitalization using biological and psychological markers.

A recent GWAS study for major depressive disorder has accentuated the need of standardized quantification of disease parameters to impede biased views on genetic architecture and underlying pathogenesis by minimal phenotyping (Cai et al. 2020). Study protocols such as the Biological Classification of Mental Disorders (BeCOME) study (Bruckl et al. 2020) focus on in depth phenotyping of patients suffering from affective, anxiety and stress-related mental disorders focusing on mental phenotypes. The present protocol complements this approach by adding somatic phenotypes under the concept of depression as a systemic disorder with a focus on cardiac phenotypes.

Preliminary analyses based on selected phenotypes in subcohorts of the GEParD and DaCFail protocols have facilitated further understanding of the disease course and risk factors in depressive episodes which will be used for comprehensive analyses of the overall study cohort and additional subgroup analyses (e.g. regarding different age groups, onset of disease manifestations):

(i) Anxious depression and traumatic events in the childhood of patients are associated with increased sensitivity of the HPA axis and the immune system using FKBP5 mRNA expression and the CTQ as a phenotypic marker (Menke et al. 2018).
(ii) Severe life events occurring prior to depressive episodes may impair psychopharmaceutical treatment assessed by FKBP5, SGK1, and NR3C1 mRNA-expression levels (Menke et al. 2021).
(iii) Covariation bias, an overestimation of the relationship between fear-relevant stimuli and aversive consequences, has been revealed as possible characterization of non-responders for antidepressant treatment. It may serve as a possible neurocognitive marker for emotional information processing in depressive episodes (Stonawski et al. 2019).
(iv) Psychological paradigms show that fear acquisition and extinction may be impaired in patients suffering from severe depressive episodes. This may underpin the importance of future studies addressing extinction learning elements in antidepressant treatment (Wurst et al. 2021).

The present two-level study design faces limitations of which the foremost are: the naturalistic and observational inpatient setting does not allow for controlled interventions with randomized and matched treatment and control samples. Comprehensive diagnostics results in a high number of single procedures. This may challenge depressed patients who are treated in an inpatient setting because of severe depression and cause a relevant dropout of patients due to motivation, distress, and wishes for discharge (Figs. 1 and 2, Tables 3, 4 and 5). In addition, this contributes to a fragmentary mosaic of quantified biomarkers with missing data, which counteracts the effort for standardized endo-phenotyping of depressive episodes in a cohort representative for the inpatient population. The Department of Psychiatry, Psychotherapy and Psychosomatic Medicine of the University Hospital of Würzburg provides specialized and acute psychiatric care for Würzburg, a medium-sized German university town, which in a monocentric approach limits the recruitment of rare, however, severely ill patient groups, such as DaCFail study group 1 (depression and HF, Fig. 2).

Overall, the described concept for deep phenotyping of depressive episodes illustrates a promising approach to unravel predictive measurements for the onset, course, and treatment response of depressive episodes with a focus on heart comorbidity using a broad repertoire of established psychometric, somatic, and laboratory including (epi-)genetic markers. Experiences with these mainly naturalistic protocols will contribute to successful multicentre studies investigating personalized antidepressant therapies.

## Data Availability

The datasets generated during the study are available from the corresponding author on reasonable request.
